# GPR30 receptor promotes preoperative anxiety-induced postoperative hyperalgesia by up-regulating GABA_A_-α4β1δ subunits in periaqueductal gray in female rats

**DOI:** 10.1186/s12871-020-01017-7

**Published:** 2020-04-22

**Authors:** Ming Jiang, Yu’e Sun, Yishan Lei, Fan Hu, Zhengrong Xia, Yue Liu, Zhengliang Ma, Xiaoping Gu

**Affiliations:** 1 Department of Anesthesiology, Affiliated Drum Tower Hospital of Nanjing University Medical School, Nanjing, 210008 Jiangsu Province China; 2 Analytical & Testing Center, Nanjing Medical University, Nanjing, Jiangsu Province China

**Keywords:** GPR30 receptor, GABA_A_-α4β1δ subunits, Preoperative anxiety, Postoperative hyperalgesia, Female rat

## Abstract

**Background:**

G-protein coupled estrogen receptor 30 (GPR30) was proved the specific estrogen receptor relating to mechanical hyperalgesia. Studies have shown that the GABA_A_ receptor subunits α4, β1, and δ in the periaqueductal gray (PAG) neurons promote the descending facilitation system. This study inquired into whether and how GPR30 and GABA_A_-α4β1δ in the PAG promote preoperative anxiety-induced postoperative hyperalgesia in female rats.

**Methods:**

All the female rats were subjected to the single prolonged stress (SPS) to stimulate preoperative anxiety. Subsequently, mechanical allodynia was evaluated before and after the incision, based on the paw withdrawal mechanical threshold (PWMT). The selective GPR30 agonist G1 and antagonist G15 were locally microinjected into the PAG. The expression of GPR30, protein kinase A (PKA), and GABA_A_ receptor subunits α4, β1, and δ in the PAG neurons were detected using western blotting and immunofluorescence.

**Results:**

Behavioral testing revealed that Group S and Group I decreased the nociceptive threshold levels of PWMT in female rats. PWMT in Group S + I decreased more than that of Group S and Group I. Further, results of western blotting showed the expression of GPR30, PKA, and GABA_A_ α4, β1, and δ subunits significantly up-regulated in Group S + I, and immunofluorescence indicated that the neurons of PAG in Group S + I appeared simultaneously immunopositive for GPR30 and GABA_A_ α4, β1, and δ receptors. After microinjection of G1 into the PAG, female rats with plantar incision continued to exhibit significant hyperalgesia until postoperative 48 h. On the other hand, microinjection of G15 with SPS and plantar incision procedure relieved postoperative hyperalgesia in female rats. Western blotting demonstrated that intra-PAG injection of G15 markedly decreased the GPR30, PKA, and GABA_A_ α4, β1, and δ levels in Group G15 + I.

**Conclusions:**

Our results indicate that the GPR30-PKA-GABA_A_α4β1δ pathway in the PAG promotes preoperative anxiety-induced postoperative hyperalgesia in female rats. This mechanism might be a potential novel therapeutic target for hyperalgesia in females.

## Background

Preoperative anxiety is a clinically significant problem, with an incidence rate of 60 to 92%, for surgical patients [1]. Previous studies have demonstrated the positive correlativity between preoperative anxiety and postoperative pain [2]. Further, preoperative anxiety is often related to a slower and more algesic postoperative recovery of patients undergoing surgery [3]. Enhanced nociception and pain sensitivity due to exposure to physical or psychological stressors is described as stress-induced hyperalgesia (SIH) [4]. Although experimental studies have been conducted to investigate this phenomenon in male animals [5], the potential underlying mechanism has not been fully elucidated in female animals.

The G-protein coupled receptor 30 (GPR30), a specific estrogen receptor localized in the cell membrane, mediates the fast, non-genomic effects of estrogen [6]. It has been shown that estrogen-induced mechanical hyperalgesia is produced by selective agonists of the GPR30 receptor, and is inhibited by knockdown of the GPR30 receptor [7]. Required for complex functions, the periaqueductal gray (PAG) is associated with anxiety, fear, pain and analgesia [8]. A recent study shows that, via the p38 MAPK signaling pathway, descending facilitation of neuropathic pain is stimulated by glial activation in the PAG [9]. Further role of the GPR30 are demonstrated by studies showing GPR30 expression in the amygdala and PAG [10]. Given that PAG plays a vital part in the descending pain pathway and emotion response, it is of great importance to explore how this structure mediates anxiety-induced allodynia in females.

Accumulating evidence has indicated that GABA_A_-mediated activation of neurons facilitates hypersensitivity in the central nervous system (CNS) [11]. Study has shown the up-regulation of GABA_A_α4 subunit is associated with anxiety disorders in the female rat [12]. It has been demonstrated that the increase in GPR30 levels under stress significantly reverses the attenuation of GABA_A_ receptor, and causes its up-regulation in the amygdala of female mice [13]. Moreover, the up-regulation of GABA_A_ subunits α4β1δ in the PAG promotes the endogenous descending facilitation system [14]. However, whether GPR30 and GABA_A_ subunits in the PAG contribute to preoperative anxiety-induced postoperative hyperalgesia in females remains unknown.

This study aims to test the hypothesis that preoperative anxiety-induced elevated GPR30 in the PAG evoked the up-regulation of extrasynaptic α4, β1, and δ GABA_A_ receptor subunits through protein kinase A (PKA), and thus exacerbated postoperative pain in female rats. We conducted the experiment in a model of preoperative anxiety-induced postoperative hyperalgesia [15], with single prolonged stress (SPS) procedure and plantar incision.

## Methods

### Laboratory animals and estrogen replacement

Laboratory Animal Centre of Drum Tower Hospital (Nanjing, China) supplied adult female Sprague-Dawley rats, weighing 250 to 300 g. In total, 6 rats were housed in one cage under a 12 h light/dark cycle at 20 °C, with a relative humidity of 55% and with free access to water and food. The experimental procedures were agreed by the Laboratory Animal Ethics Committee of the Affiliated Drum-Tower Hospital of Medical College of Nanjing University [16] (Approval number 20150603), and efforts were put forth to reduce the quantity of rats and relieve the suffering.

All the female rats received bilateral ovariectomy (OVX). According to the study by Xu [17], the OVX rats were subcutaneously injected with 17β-estradiol-3-benzoate (E2) (0.1 mg·kg^− 1^·day^− 1^) for 10 days. This replacement therapy has been demonstrated to maintain an average concentration of plasma E2 between the nadir and peak levels of normal estrous cycle in the OVX rats [18].

### Drugs and experimental grouping

Drugs were prepared and administered referring to previous studies. We used 17β-estradiol-3-benzoate (E2, Sigma, St. Louis, MO) for E2 replacement therapy. G1, (GPR30 agonist) and G15 (GPR30 antagonist) were administered for the local PAG microinjection (Cayman Chemical, Ann Arbor, MI, USA). The dosages of E2 and G1/G15 were adopted according to previous studies [18], and our preliminary experiments. The E2 was diluted in sesame seed oil, and other drugs were administered with dimethyl sulfoxide (DMSO) as the vehicle.

**Fig. 1:**
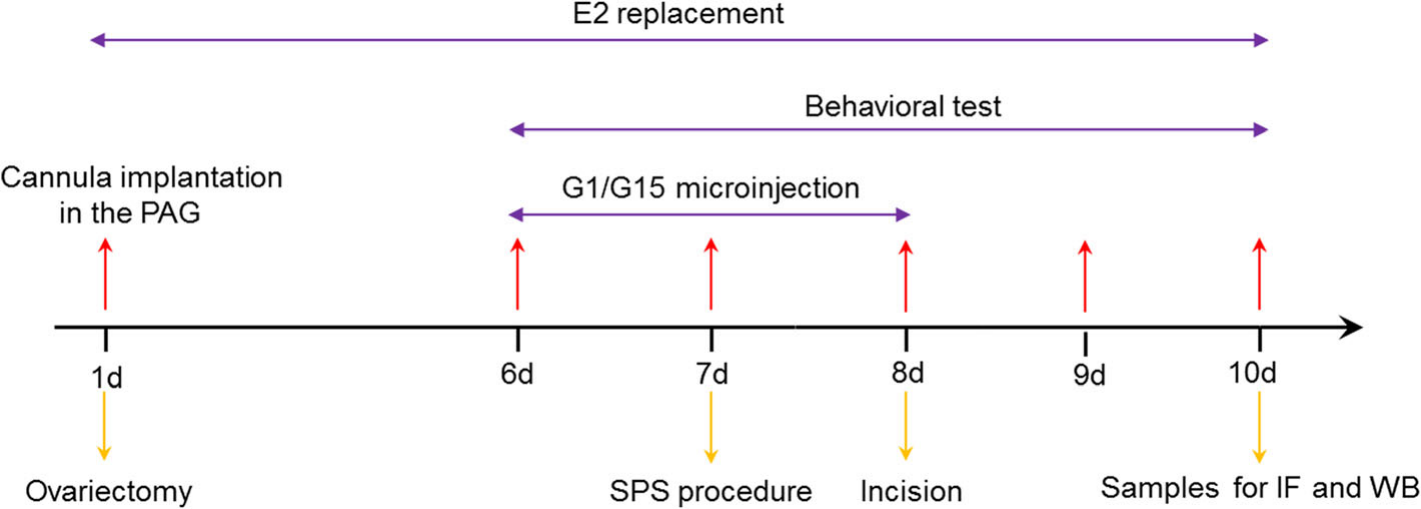
The schematic representation of the experimental design and time schedule of the protocol. All the female rats were subjected to bilateral ovariectomy and cannula implantation in the periaqueductal gray on day 1, and received estrogen replacement for 10 days. The single prolonged stress was administered on day 7, and G1/G15 microinjection was once per day from day 6 to 8 after the estrogen replacement. Behavioral tests were conducted on day 6 prior to the incision surgery, and on day 8, 9, and 10 after the surgical incision. Samples were collected for immunofluorescence and western blotting analysis on day 10 after behavioral tests

The rats were randomly divided into the 10 groups (*n* = 6 rats per group): All the female rats received bilateral ovariectomy on day 1, and subcutaneously received estrogen replacement for 10 days. Group C was a control for sham SPS and sham incision surgery experiments; Group S underwent SPS procedure on day 7; Group I was subjected to surgical incision on day 8 only; Group S + I underwent the SPS experimentation on day 7, and received incision surgery on day 8 after estrogen replacement. To investigate the effect of GPR30 in PAG, the PAG of Group G1 (10 μmol/L, 0.5 μL) and Group Vehicle1 (0.5 μL) were microinjected once per day, from day 6 to 8 without SPS and surgical incision; Group G1 + I and Group Vehicle1 + I were microinjected with the same conditions, from day 6 to 8, without SPS (30 min before surgical incision on day 8); Group G15 + I (20 μmol/L, 0.5 μL) and Group Vehicle2 + I (0.5 μL) were treated with PAG microinjection from day 6 to 8 (30 min before SPS on day 7, and 30 min before surgical incision on day 8). The procedures and treatments in 10 groups in this study are shown in Table 1, and the schematic representation of the experimental design and time schedule of the protocol are shown in Fig. 1.

**Table 1:** Procedures and treatments in 10 groups in this study

Group	Procedures and treatments
Group C	sham SPS and sham incision surgery
Group S	SPS procedure
Group I	incision surgery
Group S + I	SPS procedure and incision surgery
Group G1	PAG microinjection of G1
Group Vehicle1	PAG microinjection of Vehicle1
Group G1 + I	PAG microinjection of G1 and incision surgery
Group Vehicle1 + I	PAG microinjection of Vehicle1 and incision surgery
Group G15 + I	PAG microinjection of G15, SPS procedure, and incision surgery
Group Vehicle2 + I	PAG microinjection of Vehicle2, SPS procedure, and incision surgery

### SPS procedure and surgical incision

The SPS procedure was carried out according to the previous studies [19]. On day 7 after the estrogen replacement, female rats were confined in animal holders for 2 h, after which they were immediately forced to swim for 20 min individually in a clear acrylic cylinder (with a diameter of 30 cm and a height of 60 cm) filled with water (24 to 26 °C) to two-thirds of its height. After that, the female rats took a rest for 15 min and then exposed to the anesthetic ether until their consciousness vanished. After the animals were fully anesthetized following the administration of ether, we observed that righting reflex and corneal reflex of the rats disappeared. Control rats, untreated, were observed in another room.

The model of postoperative pain was operated on as described by Brennan [20] on day 8 after estrogen replacement. After sterilization of the right hind paw with 10% povidone-iodine, a 1 cm incision was operated on the skin and fascia. In this step, the muscle was remained intact. The skin was stitched up with 5–0 silk thread, after carefully maintaining hemostasis with pressure. Finally, the wound was covered with erythromycin ointment. The control group received sham surgery, consisting of sterilization of the hind paw, and application of erythromycin ointment on the plantar surface, without plantar incision.

### Pain-related behavioral test

To assess the pain-related behaviors in the female rats, the paw withdrawal mechanical threshold (PWMT) was measured on day 6 (24 h prior to SPS, baseline), 8, 9, and 10 (2, 6, 24, and 48 h after the incision surgery). The PWMT was evaluated by the “up-and-down” method, using a set of von Frey filaments. The OVX female rats were kept in mesh-bottomed plastic cases (20 × 20 × 15 cm) with an approximately 30 min habituation. Each von Frey filament was positioned beneath the right hind paw, and was adjacent to the wound for 6 to 8 s. Positive responses were considered as paw lifting or licking of the paw following stimulation. We repeated the stimulation 3 times, with intervals of 5 min, recorded the data, and analyzed the data using the method described by Chaplan [21].

### Intra-PAG microinjection

Rats were anesthetized with sevoflurane by a nose mask (induction, 3%; surgery, 1%; Heng Rui Co., Shanghai, China) and placed in a stereotaxic frame. After locating the bregma by exposing the skull of the rat, a small hole was drilled for cannula implantation in the PAG (bregma, − 7.8 mm, 0.6 mm laterally, and − 4.5 mm ventrally from the skull surface). The microinjection cannula was immobilized with dental cement. In the preliminary experiment, we used the stereotaxic instrument to perform multiple injections of Evans blue in the PAG of female rats to determine the correct injection site. Rats were given 5 days to recover after implantation, and body weights were measured. On the day of intra-PAG injection, the rats were left without being disturbed for 1 h before the microinjection. In this study, drugs were injected over a 2-min period, and the volume of drug administered into the PAG was 0.5 μL. To examine the active role of GPR30 in the PAG in SPS-enhanced postoperative hyperalgesia, the selective GPR30 agonist G1 and antagonist G15 were administered into the PAG by local microinjection.

### Immunofluorescence

Immunofluorescence was used to further study the female rats. The animals were anesthetized deeply with 5% sevoflurane and sacrificed by cervical dislocation. Rats were then perfused with 4% paraformaldehyde in 0.1 M phosphate buffer (pH = 7.4) on day 10 after behavioral tests. Next, the brain was removed, post-fixed in the same fixative, and transferred to 30% sucrose. The PAG tissues were cut into 25 μm-thick sections, and they were incubated overnight with the primary antibodies: rabbit polyclonal to GPR30 (Abcam, Cambridge, UK; 1:200), goat polyclonal antibody against the GABA_A_α4 receptor (N-19; Santa Cruz Biotechnology, TX, USA; 1:250), mouse monoclonal to GABA_A_ β1 receptor (S96–55; Abcam; 1:200), or goat polyclonal antibody against the GABA_A_δ receptor (R-20; Santa Cruz Biotechnology; 1:250). After washing, sections were incubated with secondary antibodies: Alexa Fluor 555-conjugated goat anti-mouse, Alexa Fluor 488-conjugated goat anti-rabbit (1:1000, ThermoFisher Scientific, MA, USA) and NorthernLights 557-conjugated donkey anti-goat (1:1500, R&D Systems, MN, USA) for 1 h at room temperature. The stained sections were mounted on glass slides, and examined under a LSM710 (Carl Zeiss, Germany) confocal microscope.

### Western blotting

The segments of PAG were removed rapidly from deeply anesthetized rats, and stored at − 80 °C. The collected tissue samples were homogenized in lysis buffer and centrifuged (4 °C) at 13,000 rpm for 10 min. Protein samples were separated using sodium dodecyl sulfate polyacrylamide gel electrophoresis (SDS-PAGE, 10%), and transferred onto a nitrocellulose membrane. The membranes were incubated overnight at 4 °C with primary antibodies against GPR30 (1:500, Abcam), PKA (1:500, R&D Systems), GABA_A_α4 (1:1000, Santa Cruz Biotechnology), GABA_A_β1 (1:1000, Abcam), and GABA_A_δ (1:1000, Santa Cruz Biotechnology). The membranes were washed and incubated with secondary antibody for 1 h at room temperature. β-actin was used as a loading control. The immune complexes were detected using enhanced chemiluminescence. Density of specific bands was analyzed using Image-Pro Plus software.

### Statistical analysis

All data were expressed as mean ± standard deviation (S.D.). Two-way repeated measures analysis of variance (ANOVA) was performed to analyze differences in pain-related behaviours. Bonferroni post-hoc tests were conducted to determine the source(s) of differences when significant main effects were observed. Kruskal-Wallis test was used for comparisons of the non-parametric data regarding western blot. All statistical analyses were performed using SPSS 17.0 software (SPSS Inc., Chicago, IL, USA), and *P* ≤ 0.05 was set as the level of statistical significance.

## Results

### Preoperative SPS-induced postoperative hyperalgesia in female rats

**Fig. 2:**
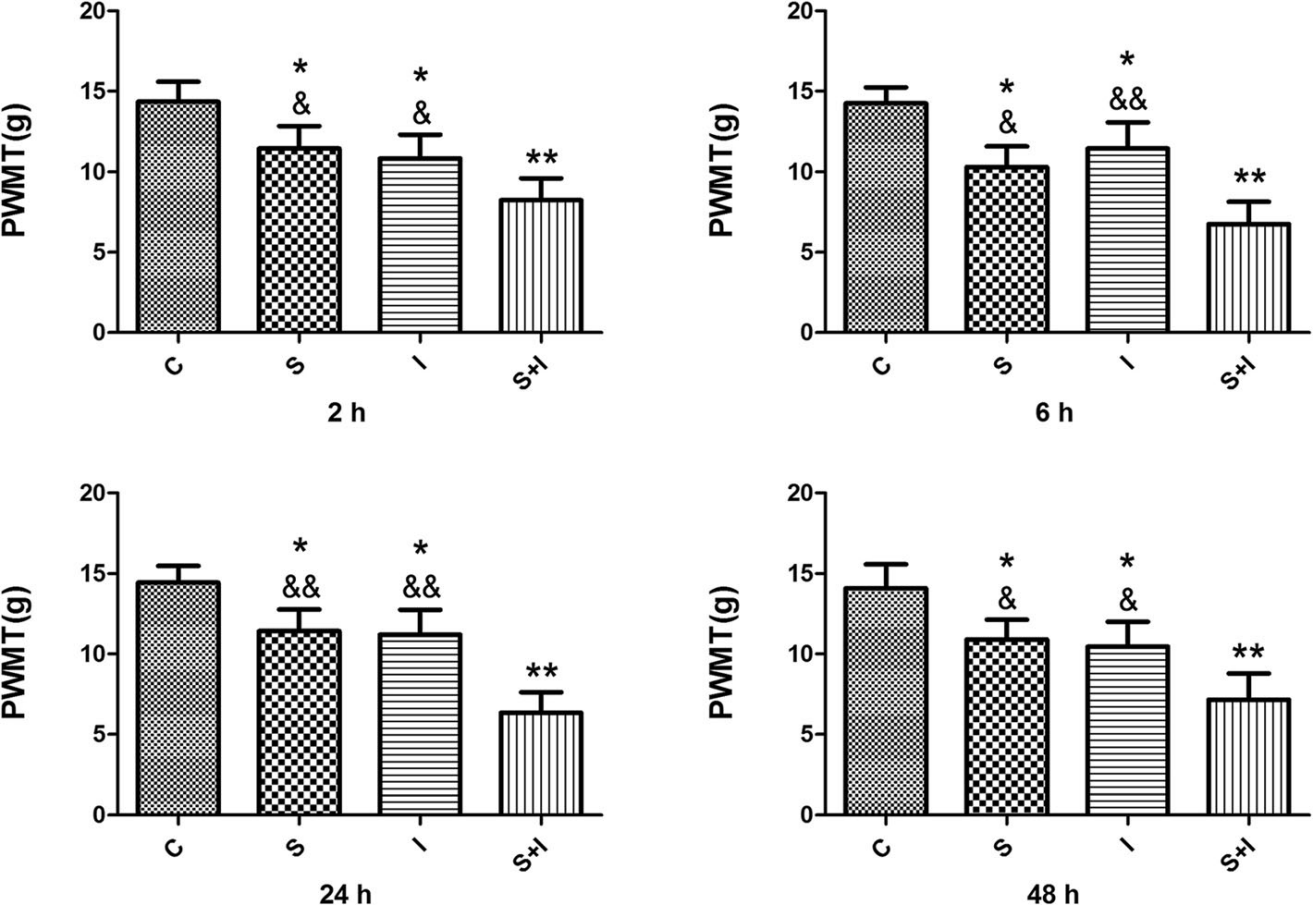
Paw withdraw mechanical threshold (PWMT) was examined at 2 h, 6 h, 24 h and 48 h after the plantar incision (*n* = 6/each group). Decrease PWMT in Group S, Group I and Group S + I were observed at each time-point after the incision. ^*^P < 0.05, ^**^P < 0.01 compared with Group C; ^&^*P* < 0.05, ^&&^*P* < 0.01 compared with Group S + I

The baseline PWMT was not significantly different of PWMT across groups on day 6 before incision surgery. PWMT in Group S, Group I and Group S + I were observed significantly lower than Group C at each time-point from day 8 to 10 (Fig. 2). SPS and surgical incision in Group S + I significantly enhanced mechanical allodynia compared to the Group S. Meanwhile, the PWMT in Group S + I significantly decreased compared to the Group I from day 8 to 10.

### Up-regulation of GPR30 and GABA_A_α4β1δ in the PAG of female rats

**Fig. 3:**
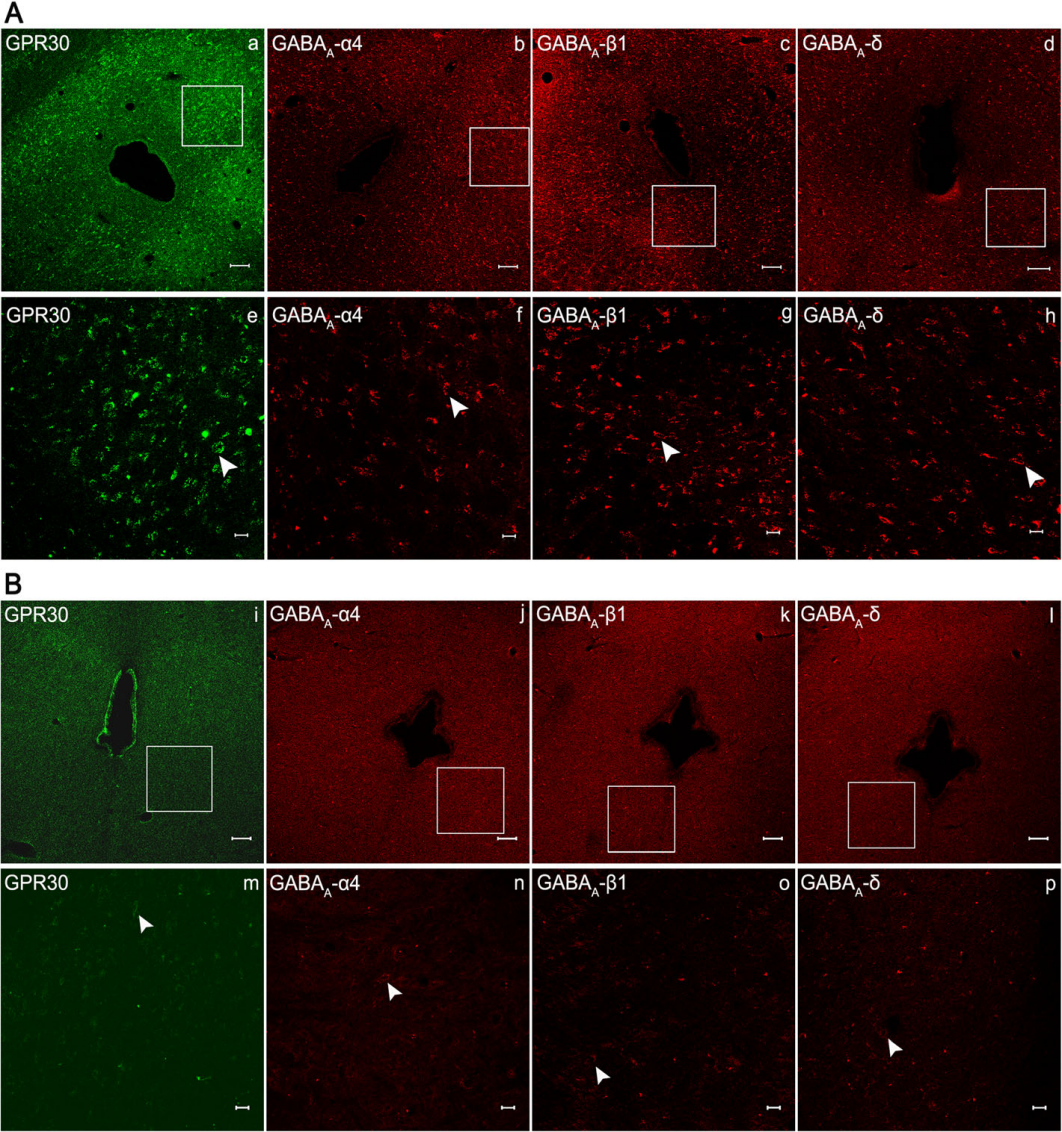
Confocal fluorescence micrographs of the periaqueductal gray (PAG) neurons with the immunolabeled GPR30 (a, e, i, m), GABA_A_α4 (b, f, j, n), GABA_A_β1 (c, g, k, o), and GABA_A_δ (d, h, l, p) receptors. **a** Representative images of Group S + I. **b** Representative images of Group I. Scale bar is 100 μm in a–d and i–l, and 20 μm in e–h and m–p

**Fig. 4:**
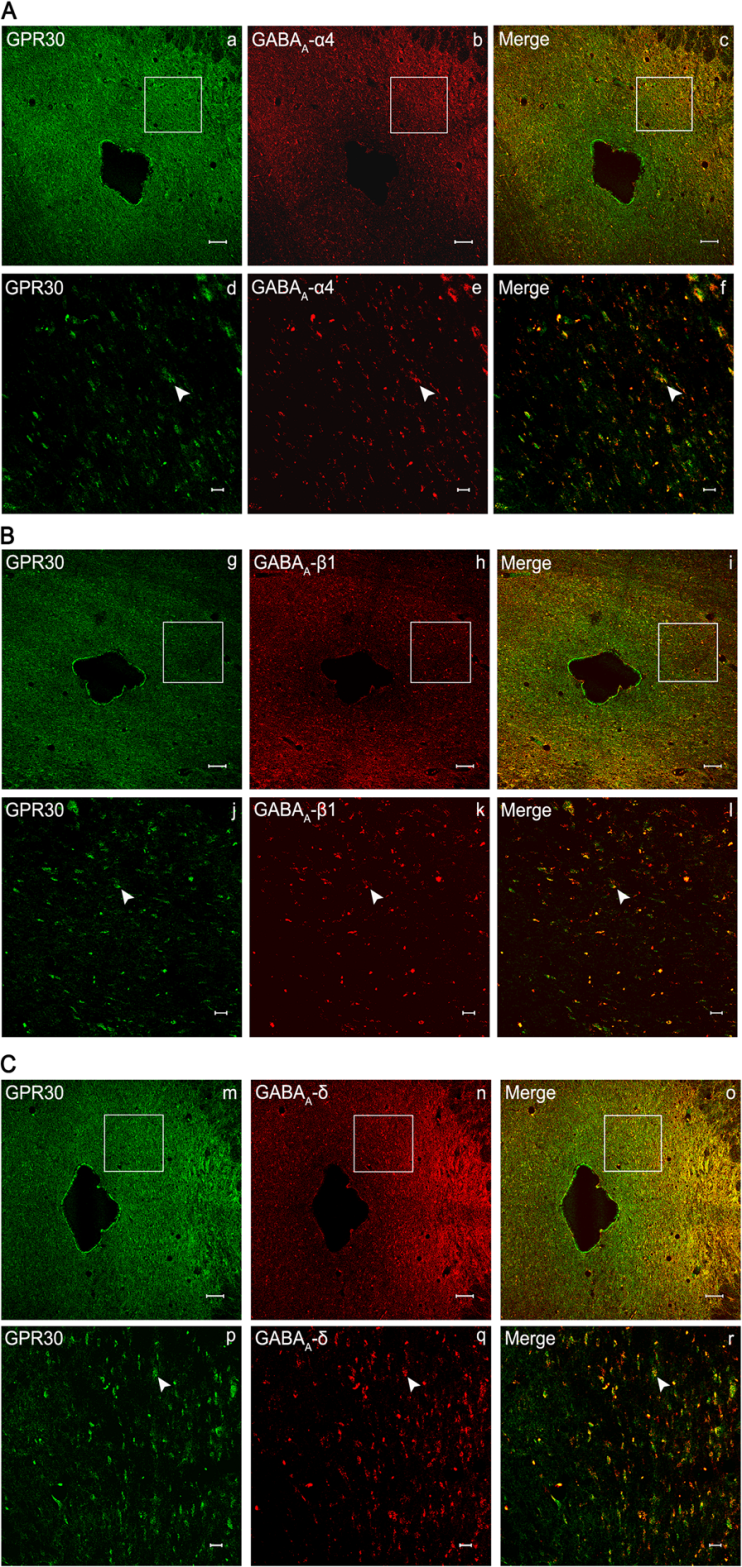
Co-expression of GPR30 and GABA_A_α4 (**a**), GPR30 and GABA_A_β1 (**b**), as well as GPR30 and GABA_A_δ (**c**) in periaqueductal gray (PAG) after incision surgery. Double immunofluorescence staining for confocal laser scanning microscopy showing a transverse section of PAG. In Group S + I, GPR30/GABA_A_α4 (c, f), GPR30/GABA_A_β1 (i, l), and GPR30/GABA_A_δ (o, r), simultaneously immunopositive in neurons in PAG were co-localized, as indicated by yellow overlay. Scale bar is 100 μm in a–c, g–i, and m–o, and 20 μm in d–f, j–l, and p–r

The neuronal receptors of PAG were observed under the confocal microscope. The receptors GPR30 and GABA_A_ α4, β1, and δ were expressed in the PAG of rats in Group S + I (Fig. 3a) and Group I (Fig. 3b). Double immunofluorescence staining for co-expression of GPR30 and GABA_A_α4, GPR30 and GABA_A_β1, as well as GPR30 and GABA_A_δ were observed in Group S + I (Fig. 4a–c). The results showed that GPR30/GABA_A_α4 (Fig. 4a), GPR30/GABA_A_β1 (Fig. 4b), and GPR30/GABA_A_δ (Fig. 4c), simultaneously immunopositive in neurons in PAG were co-localized, as indicated by yellow overlay.

**Fig. 5:**
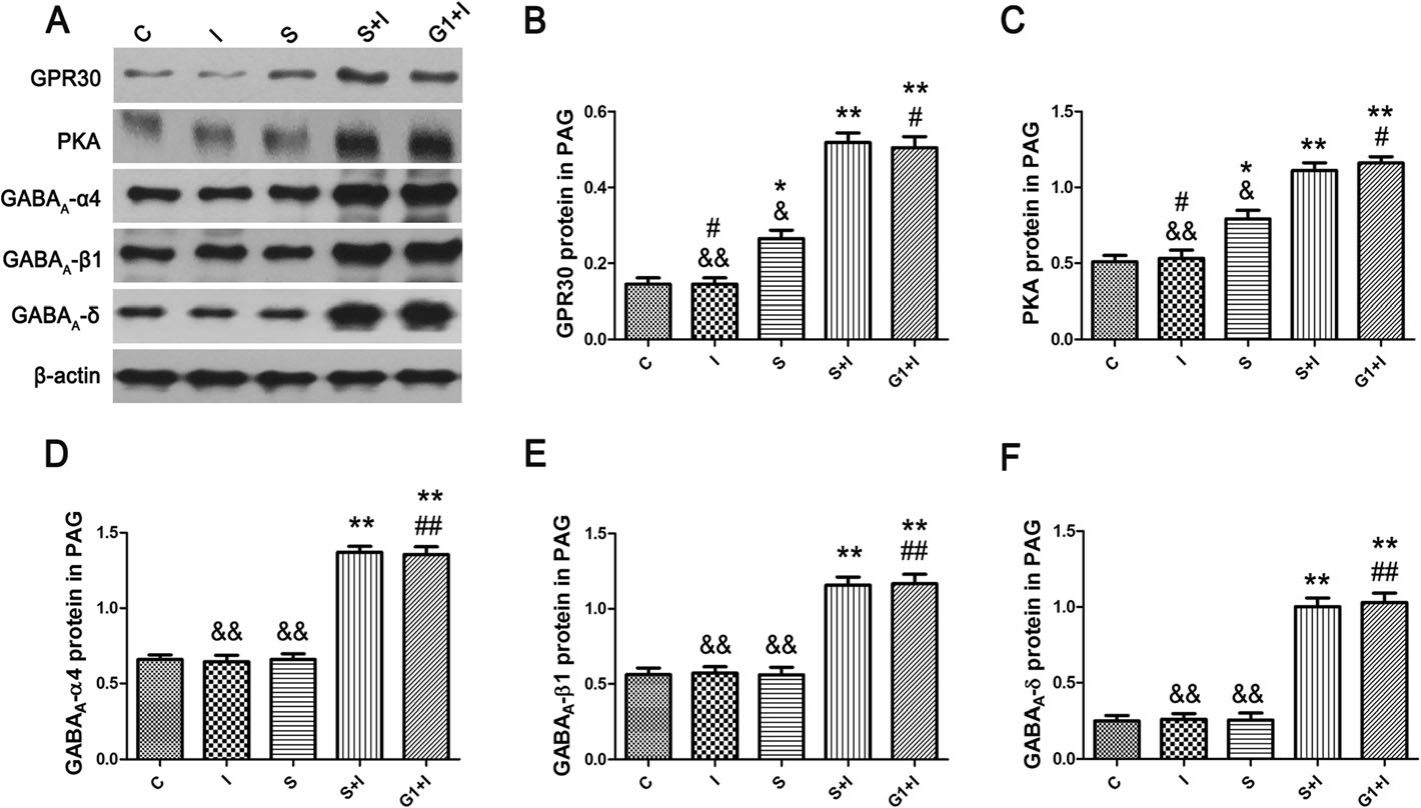
Western blotting of GPR30, PKA, and GABA_A_ α4, β1, and δ subunits in periaqueductal gray neurons of different groups (*n* = 6/each group). β-actin was used as a loading control. The expressions of GPR30 and PKA in Group S + I and Group G1 + I were significantly higher than in other groups (**a-f**). The expression level of GPR30 and PKA in Group S was higher than in Group C. ^*^P < 0.05, ^**^P < 0.01 compared with Group C; ^#^*P* < 0.05, ^##^*P* < 0.01 compared with Group S; ^&^*P* < 0.05, ^&&^*P* < 0.01 compared with Group S + I

Western blotting demonstrated that the expression of GPR30, PKA, and GABA_A_ α4, β1, and δ of Group S + I rats were significantly higher than that of other groups (Fig. 5a-f). The expression level of GPR30 and PKA in Group S was higher than in Group C. Female rats that received SPS without incision surgery showed higher levels of GPR30 and PKA, but the levels of GABA_A_ receptors were not different from those of Group C. Moreover, there were no significant changes in the levels of GPR30, PKA, and GABA_A_ receptors between Group I and Group C. Furthermore, we compare the female rats only exposed to the anesthetic ether according to the SPS procedure with the control rats without the ether, and no significant changes were found in the levels of GABA_A_-α4β1δ subunits between the two groups.

### Effects of GPR30 in PAG for SPS-enhanced postoperative allodynia in female rats

**Fig. 6:**
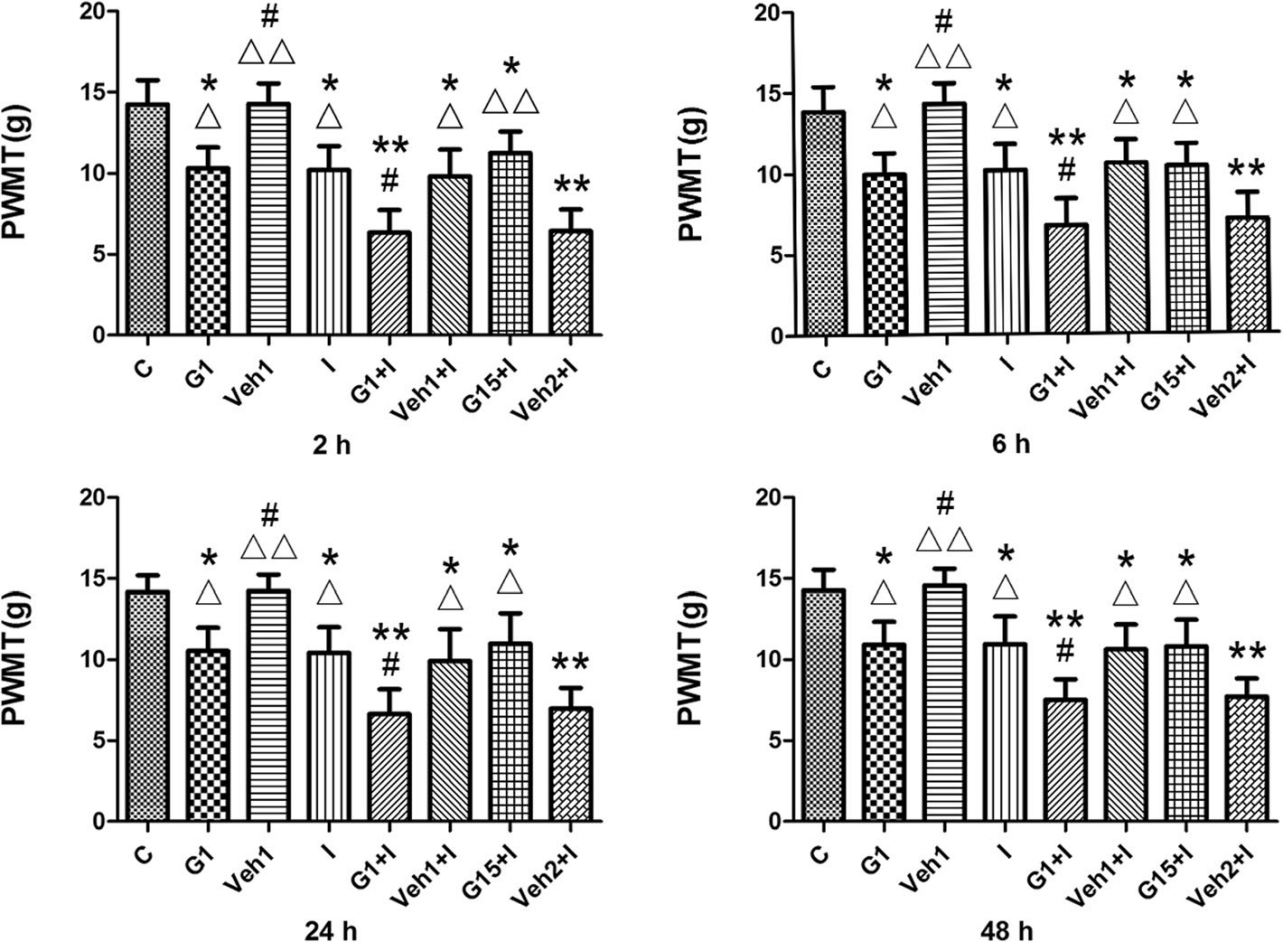
Paw withdraw mechanical threshold (PWMT) was examined at 2 h, 6 h, 24 h and 48 h after the plantar incision (n = 6/each group). Decreases in PWMT were observed in Group G1, Group I, Group G1 + I, Group Veh1 + I, Group G15 + I and Group Veh2 + I at each time-point after the incision. ^*^P < 0.05, ^**^P < 0.01 compared with Group C; ^#^*P* < 0.05 compared with Group I; ^△^*P* < 0.05, ^△△^*P* < 0.01 compared with Group Veh2 + I

Our results showed no significant difference in PWMT between the sham and model groups prior to incision surgery. Decreases in PWMT were observed in Group G1, Group I, Group G1 + I, Group Veh1 + I, Group G15 + I and Group Veh2 + I at each time-point after the incision (Fig. 6). A decreased tolerance to nociceptive stimulus was observed in Group G1 compared with Group C from 2 h to 48 h. Furthermore, in Group G1 + I, intra-PAG microinjection of G1 significantly declined in nociceptive thresholds after the surgical procedure compared with Group I. No significant changes were found in PWMT between Group G15 + I and Group I, while the threshold levels of Group G15 + I were relatively higher than those of Group Veh2 + I. Moreover, there were no significant differences in the PWMT between the groups receiving vehicle and the groups undergoing the same procedure without DMSO (Fig. 6).

**Fig. 7:**
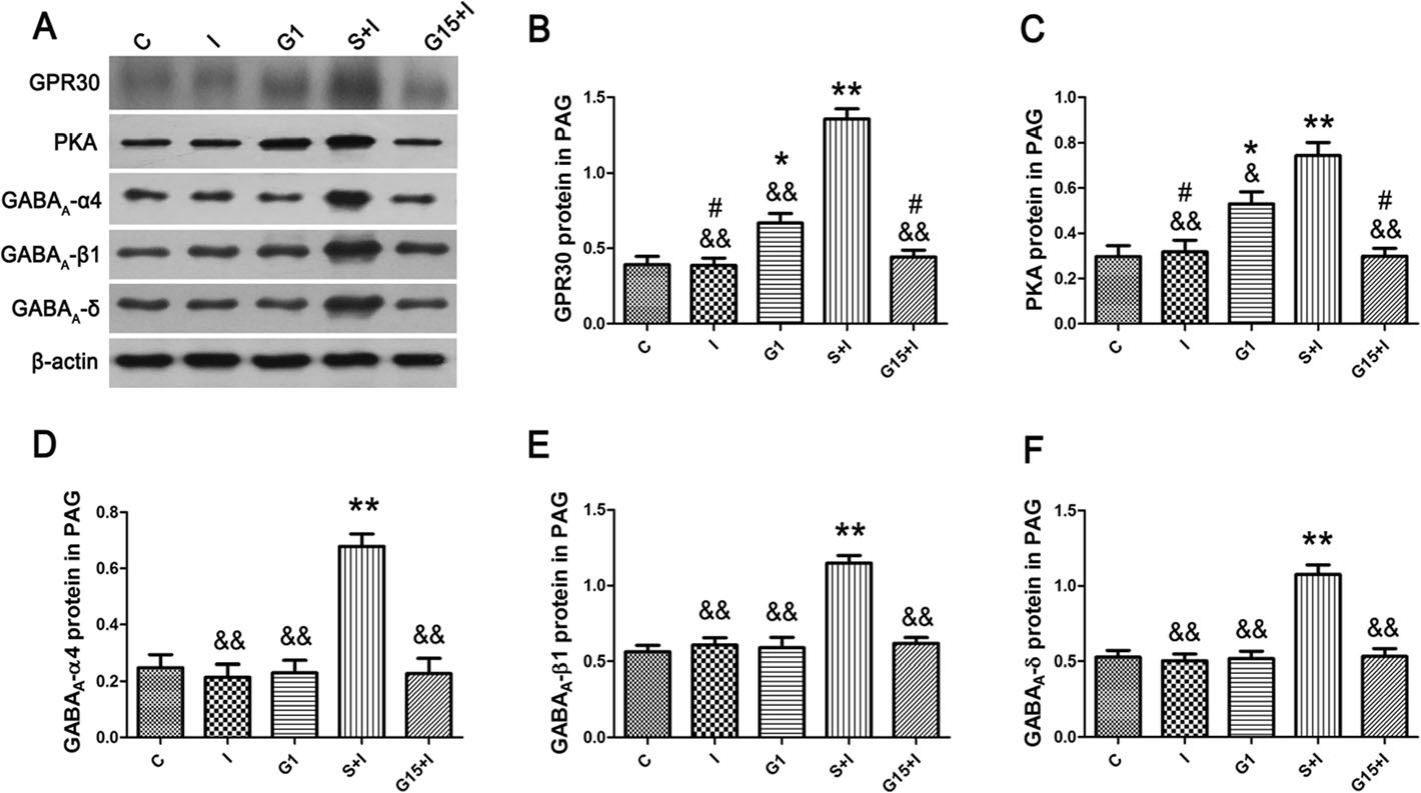
Western blotting of GPR30, PKA, and GABA_A_ α4, β1, and δ subunits in periaqueductal gray neurons of different groups (n = 6/each group). β-actin was used as a loading control. The expressions of GPR30 and PKA in Group G1 and Group S + I were significantly higher than in other groups (**a-f**). Further, Group S + I showed higher intensity of the GPR30 and PKA than Group G1. ^*^P < 0.05, ^**^P < 0.01 compared with Group C; ^#^*P* < 0.05 compared with Group G1; ^&^*P* < 0.05, ^&&^*P* < 0.01 compared with Group S + I

**Fig. 8:**
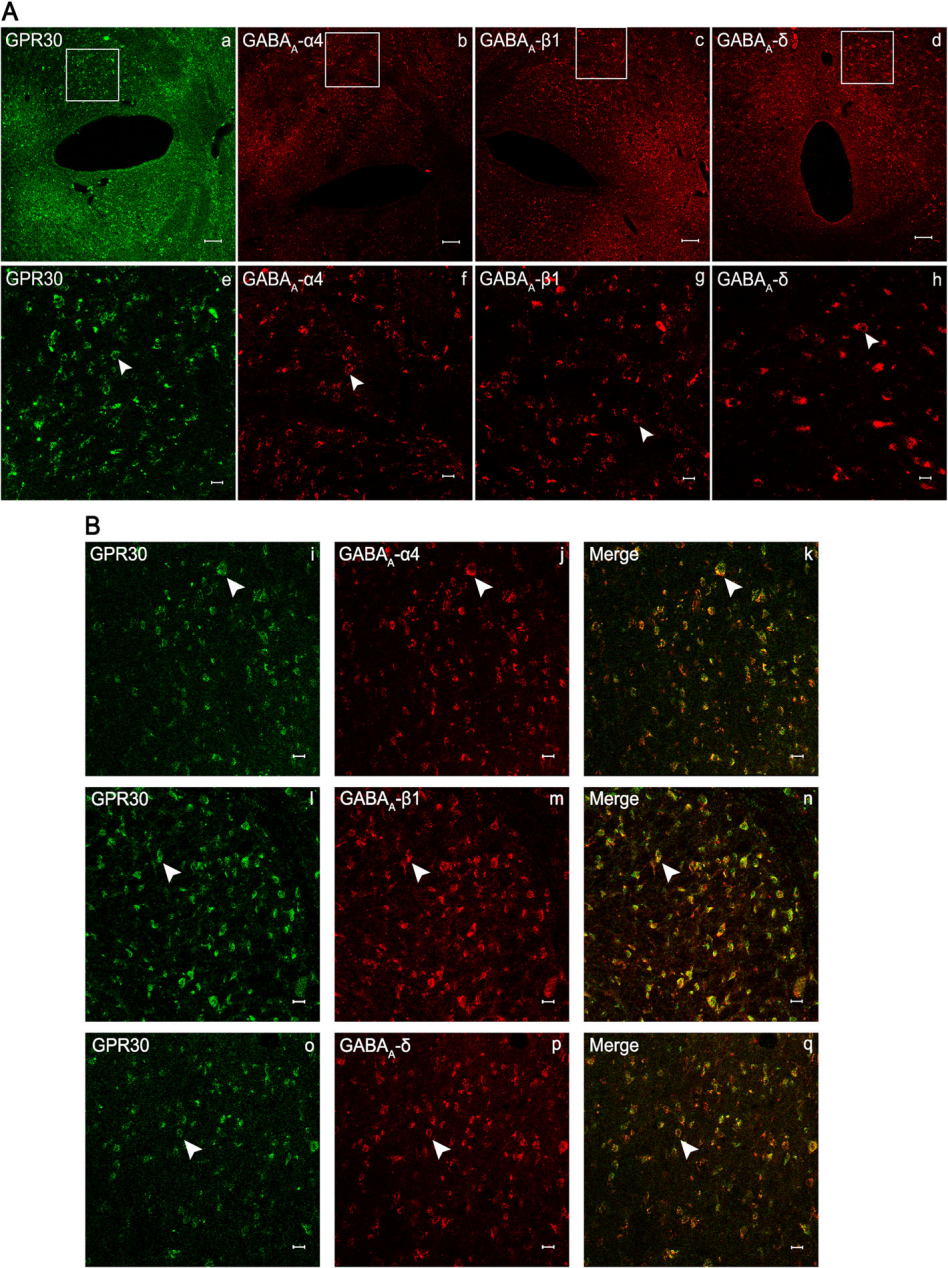
Confocal fluorescent micrographs of the periaqueductal gray (PAG) neurons immunolabeled GPR30 and GABA_A_ α4, β1, and δ subunits in Group G1 + I (**a**). GPR30/GABA_A_α4, GPR30/GABA_A_β1, and GPR30/GABA_A_δ of neurons in PAG were co-localized, as indicated by yellow overlay (**b**). Scale bar is 100 μm in a–d and 20 μm in e–q

Results from the western blotting showed that microinjection of G1 in the PAG significantly up-regulated the expression of GPR30, PKA, and GABA_A_ α4, β1, and δ after incision operation compared with the Group C, but no significant difference was found compared with Group S + I (Fig. 5). The expressions of GPR30 and PKA in Group G1 and Group S + I were significantly higher than in other groups (Fig. 7). Further, Group S + I showed higher intensity of the GPR30 and PKA than Group G1. In Group G1, the expression of GPR30 and PKA significantly increased, but no significant difference of GABA_A_ α4, β1, and δ was found compared with Group C. In contrast, intra-PAG injection of G15 markedly decreased the GPR30, PKA, and GABA_A_ α4, β1, and δ levels in the PAG compared with Group S + I (Fig. 7). Additionally, the staining expression of GPR30 and GABA_A_ α4, β1, and δ in the PAG were observed in Group G1 + I, which was microinjected with G1, and underwent incision surgery (Fig. 8a). Double immunofluorescence images of the PAG showed the co-expression of GPR30 and GABA_A_ α4, β1, and δ in the rats of Group G1 + I (Fig. 8b).

## Discussion

In this study, we demonstrated (1) that preoperative SPS-induced postoperative hyperalgesia in female rats via up-regulation of GPR30, PKA and GABA_A_α4β1δ in the PAG, (2) the previously unknown effects of GPR30 on SPS-induced postoperative allodynia in PAG of female rats with incisions, (3) microinjection of G1 in the PAG continued to exhibit significant hyperalgesia in female rats with plantar incision, and microinjection of G15 with SPS and plantar incision procedure relieved postoperative hyperalgesia in female rats. These findings support the hypothesis that upregulation of neuronal GPR30-PKA-GABA_A_α4β1δ pathway in the PAG promotes preoperative anxiety-induced postoperative hyperalgesia in female rats.

Among patients undergoing surgery, preoperative anxiety has a very high incidence [1], which is related to acute postoperative pain, and even the chronic postoperative pain [22]. However, only a few studies have been conducted to investigate this phenomenon in females. The PAG, a core component of the descending pain-modulatory network, is a pre-eminent central trigger site for behavioral and pain transmission via the rostral ventromedial medulla (RVM), projecting to the spinal dorsal horn [23, 24]. The PAG has been demonstrated to be associated with vibration stress-induced reduction in tail flick latency [25], and spinal nerve ligation in mice exposed to SIH results in an increase in glial fibrillary acidic protein mRNA expression in the PAG [26]. In this study, the OVX female rats with E2 replacement using SPS, and incision surgery, exhibited postoperative hyperalgesia. Taken together, these data suggest that the GPR30 up-regulates extrasynaptic GABA_A_ α4, β1, and δ subunits in the PAG neurons, and makes a difference in the progress of preoperative anxiety-induced postoperative hyperalgesia in female rats.

As a novel estrogen receptor, 7-transmembrane spanning G protein-coupled receptor, GPR30 is located in the plasma membrane, endoplasmic reticulum, Golgi complexes, and the physiological functions of GPR30 have been described in almost every organ [27]. Current evidences suggest GPR30 expression in multiple regions of the CNS in rodents (e.g. cortex, hypothalamus, hippocampus, amygdala, and PAG) [10, 28]. Further, 17β-estradiol produced GPR30-dependent primary hyperalgesia [29], which was restrained with antagonist or knockdown of GPR30. Estrogen binding with GPR30 causes the dissociation of heterotrimeric G proteins to activate intracellular signaling cascades. The Gα subunit of GPR30 initiates cyclic adenosine monophosphate (cAMP) production [30]. The Gα subunit cascades can activate the adenylyl cyclase (AC) enzymes which catalyze the conversion of adenosine triphosphate (ATP) to cAMP [31], and the Gα subunit also mobilizes calcium stores in a phospholipase C dependent manner. The rapid rise of the cAMP and Ca^2+^ concentration caused by GPR30 can ultimately lead to gene regulation and other related cellular responses [32]. We found that preoperative SPS induced the expression of GPR30 and PKA in the PAG of the female rats. Microinjection of G1 into PAG significantly decreased the nociceptive threshold after the incision operation, and female rats treated with the antagonist G15 in the PAG alleviated hyperalgesia after incision surgery. Consistent with the behavioral tests, the expression of GPR30 and PKA were significantly increased in the process of postoperative hyperalgesia.

Generally, extrasynaptic GABA_A_ receptors contain α4–6 subunits, together with either a γ2 or δ subunit, and mediate tonic inhibition [33]. It is acknowledged that the PAG is a source of descending facilitation and inhibitory pathways exerting paralleled but opposite influences on nociceptive transmission via the descending projections from the RVM to the dorsal horn [34]. The facilitation and inhibitory systems are both considered to be tonically active, and it has been indicated that a small net facilitation influence predominates under non-stressed conditions [35]. Studies have shown that the up-regulation of extrasynaptic GABA_A_ receptors with the α4, β1, and δ subunits expressed on neurons in the PAG probably inhibit the endogenous descending inhibitory system, and promote the descending facilitation system function [14]. In this study, beside GPR30 and PKA, GABA_A_ α4, β1, and δ subunits were also highly expressed in the PAG during preoperative anxiety-induced postoperative hyperalgesia. It has been shown that the expression of extrasynaptic GABA_A_ α4 subunit in cultured cortical neurons can be enhanced by PKA activation, and simultaneous exposure to a PKA inhibitor can block this effect [36]. Moreover, ethanol modulation of extrasynaptic GABA_A_ α4 and δ subunits is PKA-dependent, and PKA directly affects the expression and function of these receptors, which increase the tonic current in cortical neurons after exposure to a PKA activator, but PKC activation exerts no influence in the tonic current [37]. Aripiprazole increases the expression of GABA_A_ (containing β-1 subunit) receptor in the nucleus accumbens of rats, which is significantly correlated with the enhanced PKA signaling [38]. In addition to hyperalgesia in behavioral tests, we found that the expression (based on western blotting) of GABA_A_ α4, β1, and δ subunits significantly increased in the PAG of Group S + I and Group G1 + I, and the neurons of the PAG also appeared simultaneously immunopositive for GPR30 and GABA_A_ α4, β1, and δ subunits. However, the levels of these receptors did not increase in the PAG of Group I and Group G15 + I. We speculate that the dissociation of Gα subunit of GPR30 through cAMP enhances PKA activation in female rats after SPS, and enhances the tonic currents caused by the extrasynaptic GABA_A_ subunits α4, β1, and δ via PKA in the PAG, inhibiting the pain inhibitory system and activating the descending facilitation system, which decreases the excitation threshold of nociceptive neurons and causes postoperative hyperalgesia. Interestingly, although GPR30 and PKA expression increased in the female rats which only received the SPS, GABA_A_ receptor subunits (α4, β1, δ) protein levels were not significantly different from Group C. The reason for this phenomenon might be that a certain degree of increase in GPR30 and PKA may not up-regulate the extrasynaptic GABA_A_ α4, β1, and δ subunits without incision surgery. SPS without surgical incision were relatively lower in nociceptive thresholds than those of Group C might be through other downstream signaling pathways of GPR30-PKA system. Further in-depth studies are still needed to explore the detailed mechanisms. The limitation of our study is that not all groups were analyzed using immunofluorescence staining, and the changes in GPR30 and GABA_A_ receptor subunits were only compared between different groups using western blotting. This limitation may affect the results in our study.

Overall, these findings might be relevant for understanding of the potential mechanisms involved, and for exploring the effective prevention and treatment for this clinical issue in females.

## Conclusion

Together, the results further extend our understanding of the functional relevance of the GPR30-PKA-GABA_A_α4β1δ pathway in the PAG promoting preoperative anxiety-induced postoperative hyperalgesia in female rats. The presence of GPR30 in the PAG is an important condition for the development of preoperative anxiety-induced postoperative allodynia.

## Data Availability

The datasets used and/or analyzed during the current study are available from the corresponding author on reasonable request.

## Abbreviations

AC: Adenylyl cyclase
ANOVA: Analysis of variance
ATP: Adenosine triphosphate
cAMP: Cyclic adenosine monophosphate
CNS: Central nervous system
DMSO: Dimethyl sulfoxide
E2: 17β-estradiol-3-benzoate
ERα: Estrogen receptor-alpha
ERβ: Estrogen receptor- beta
GPR30: G-protein coupled estrogen receptor 30
OVX: Ovariectomy
PAG: Periaqueductal gray
PKA: Protein kinase A
PWMT: Paw withdrawal mechanical threshold
RVM: Rostral ventromedial medulla
SIH: Stress-induced hyperalgesia
SPS: Single prolonged stress
